# The Epistemic Imperialism of Science. Reinvigorating Early Critiques of Scientism

**DOI:** 10.3389/fpsyg.2020.609823

**Published:** 2021-01-07

**Authors:** Lucas B. Mazur

**Affiliations:** ^1^Jagiellonian University, Kraków, Poland; ^2^Sigmund Freud Privatuniversität Berlin, Berlin, Germany

**Keywords:** science, scientism, positivism, postmodernism, sapientia, wisdom, epistemology

## Abstract

Positivism has had a tremendous impact on the development of the social sciences over the past two centuries. It has deeply influenced method and theory, and has seeped deeply into our broader understandings of the nature of the social sciences. Postmodernism has attempted to loosen the grip of positivism on our thinking, and while it has not been without its successes, postmodernism has worked more to deconstruct positivism than to construct something new in its place. Psychologists today perennially wrestle to find and retain their intellectual balance within the methodological, theoretical, and epistemological struggles between positivism and postmodernism. In the process, pre-postmodern criticisms of positivism have been largely forgotten. Although they remain deeply buried at the core of psychology, these early alternatives to positivism are rarely given explicit hearing today. The current piece explores some of the early critiques of positivism, particularly of its scientism, as well as early suggestions to tip the scales (back) in favor of *sapientia* (“wisdom”). This third option, largely overlooked within mainstream psychology, is of tremendous value today as it is both deconstructive and constructive relative to the shortcomings of positivism. It avoids the overly reductionistic “trivial order” of positivism, as well as the deeply unsatisfying and disorienting “barbaric vagueness” of postmodernism, while simultaneously embracing important core elements of both currents of thought.

## Introduction

Psychological science is – for pragmatic considerations – mostly oriented toward positivism, which is perceived as a modern but not postmodern epistemology. Postmodern psychology has not yet arrived in mainstream psychology. […] How can psychology move on from modern toward a postmodern science, and is this really necessary or useful? (Frontiers in Psychology, n.d.)

These lines from the call for papers are helpful for framing the arguments made in the text below as they point to several important characteristics of contemporary psychology. It has been long-clear that, while *as a methodology* positivism is powerful, *as a philosophy* for the social sciences it is deeply problematic (Sheen, 1934/2019). By taking the limits of method to be the limits of knowledge, we have artificially narrowed our understanding of knowledge. By way of the processes of conceptualization and operationalization that grant it its power, positivistic epistemology artificially limits the person (in its *Menschenbild*) to the empirical and measurable, and disregards or even denies all else. Positivism in psychology has been linked with our “almost neurotic need to be seen as scientific” and “to reject the subjective world” (Baker, 1992, p.13). The attempt to more thoroughly describe or define positivism has been called a task taken up by the “presumptuous and masochistic” (Caldwell, 1994, p.1), a tongue and cheek expression of a sentiment found in several classic explorations of positivism, such as that by Mill (1865/2005) and Kołakowski (1968). Kołakowski (1968) argues that all such attempts are at least partially arbitrary, but that this arbitrariness is unavoidable if we are to meaningfully work with the term at all. Such an approach recognizes that within the history of positivism, “repetitions can take deceptively different forms” (Tolman, 1992, p.8). Thus, following the lead of such earlier scholarship, we will use a broad, inclusive understanding of positivism, a school of thought generally involving phenomenalism and the rejection of metaphysics, nominalism anchored in (usually quantifiable) empirical data, claims to value-free objectivity, and claims that science can be thought of as a (largely singular) enterprise that develops over time and that represents not *a* way of knowing the world but as *the* way of doing so (Kołakowski, 1968). The exact nature of this “temper of mind” or “style of thinking, which as a rule is not dealt with by its adherents” (Kołakowski, 1968, p.vi), can be “better known through the enemies of that mode of thinking than through its friends” (Mill, 1865/2005, p.1). Thus, we stand to gain a better appreciation of positivism by examining one of its opponents, postmodernism.

Postmodernism can be broadly understood as an attempt to reemphasis precisely those elements of our lives that tend to be marginalized or overlooked within positivistic epistemology (Hicks, 2011). Nevertheless, despite the best efforts of postmodern thinkers to tear down positivism, despite the label of positivism being disavowed by most and even seen as “perverse” by many psychologists today (Smythe, 1992), and despite increasingly evident epistemological and practical shortcomings of positivistic epistemologies (e.g., as seen in the “replication crisis” in psychology), positivism remains on center stage in mainstream psychology, teetering between being a philosophy and a methodology (Huniche and Sørensen, 2019). While the label of positivism is rarely explicitly used today, and even less so as a form of self-identification, exploring its influence on current psychology is in no way to “kick a dead horse,” as the expression goes, precisely because the “horse is far from dead. Positivist thinking is too powerful, even today, to go away by itself” (Tolman, 1992, p.7). Steinmetz (2005) colorfully expressed the continued presence of positivism within various disciplines of the human sciences, including psychology, as follows: “Despite repeated attempts by social theorists and researchers to drive a stake through the heart of the vampire, the disciplines continue to experience a positivistic haunting” (p.3; see also Tolman, 1992; Laudan, 1996). Thus, regardless of who we see as the hero of the story (a pertinent question itself these days given the popular love of vampire stories), we seem to be at an impasse.

Rather than focusing on the points of conflict between positivism and postmodernism, in the current piece it is argued that we should take a step back so as to examine earlier, pre-postmodern objections to positivism and that in doing so we may identify a path or paths out of the apparent stalemate; paths that include core elements of both positivism and postmodernism. Thus, the arguments presented below are not new. They are, in fact, quite old. It will be argued that the way forward is based on old questions and old insights, and thus, this piece is what we might call a reminiscence. The answer to the problems of positivism lies not in a greater embrace of postmodernism, but in a more thorough examination of our older intellectual, if not academic, roots that are critical of positivistic *philosophy*. Rather than addressing the shortcomings of positivistic epistemology (i.e., method-turned-philosophy) by means of postmodernism, the field would be better served by deeper, more consequential reflections on *sapientia*, a form of metaphysical wisdom “that has seemed to grow so weak in the modern era, as *homo sapiens* has waned and *homo sciens* has waxed” (Aeschliman, 1983, p.20). To borrow the language of Whitehead (cited in Aeschliman, 1983, p.69), this classic third option avoids the overly reductionistic “trivial order” of positivism, whereby meaningfulness is sacrificed for mathematical precision, as well as the deeply unsatisfying and disorienting “barbaric vagueness” of postmodernism, whereby meaningfulness is lost in a world of subjectivity and relativity. At the same time, *sapientia* nevertheless embraces important core elements of both currents of thought.

We begin by examining the rise of materialistic philosophies in the 17th and 18th century, which led to the spread of positivism in the 19th century. In these periods, science was in the process of breaking away from other avenues of knowledge (e.g., the arts, theology), even though many scientists themselves remained deeply committed to them (Harrison, 2015). As materiality and quantification increasingly replaced spirit and quality as the means of validity, method came to constitute a philosophy in itself (Sheen, 1934/2019). Over time, the belief in the value of scientific methodology became *scientism*, the belief in science not only as *a* philosophical school of thought but as *the* philosophical school of thought. We then briefly explore how attempts to free the social sciences from the reductionism of positivistic philosophy took the form of postmodernism. While asserting the greater complexities of our lived experiences than can be seen based on positivism alone, postmodernism is primarily a deconstructive, reactive process, unable to truly free us from positivistic reductionism. Postmodernism has highlighted the shortcomings of scientism, and fought tooth and nail against it, but it is unable to move us forward on its own two feet. It is for this reason, as reflected in the call for papers, that psychology remains largely wedded to a modern epistemology that is simply deeply unsatisfying; we have developed an epistemological Stockholm syndrome, whereby we claim to be fleeing from a philosophy we simultaneously actively profess. Finally, we examine a handful of pre-postmodern positions based on *sapientia* that have been buried under the weight of our predominantly positivistic worldview. These positions can be reemphasized in a psychology that asserts the wholeness of the human, including the material and quantifiable, but also those parts beyond the conceptual reach of the scientific method (Mazur and Watzlawik, 2016; Mazur, 2017). This third path asserts the power of science *as method*, while also *critically* and *cautiously* supporting the polyvalence and complexities of life highlighted in postmodern thought. By reasserting the primacy of metaphysics over methods, *sapientia* promotes the power of science without overextending it into philosophy, and it thereby encourages the kind of fundamental, agentic judgment and discrimination that can allow us to benefit from the insights of postmodernism without fear of being consumed by its “barbaric vagueness.”

## The Epistemic Imperialism of Science and the Rise of Scientism

“The epistemic imperialism of science” is a phrase used by Harrison (2015, p.190) to describe how science has come to be the dominant arbiter of what counts as genuine knowledge in the modern West. The empire of science was built over the last three centuries, but the true reach of its empirical aspirations only became clear relatively recently. Despite how recent science is in its modern form, it has been argued that earlier schools of thought constitute forerunners of the notion of science-as-philosophy. For example, pelagianism, the assertion that humans can reach a state of perfection by their own means without the help of God, has been pointed to as an early forerunner of positivism (Sheen, 1934/2019). Within psychology itself, the work of Ferdinand Ueberwasser (1752–1812) has been pointed to as a positivist project predating Wundt’s famous laboratory by roughly a century (Schwarz and Pfister, 2016). Such earlier intellectual roots aside, what we now perceive to be a fundamental and eternal conflict between scientific and metaphysical epistemologies (including “faith-based” epistemologies) is a relatively recent development, and it would have been completely foreign to most people, including practitioners of empirical science, in the 17th, 18th, and even into the 19th centuries (Hunter, 2000; Harrison, 2015). An important element in the development of this modern epistemic rift is the emergence of the modern notion of objectivity, whereby validity is thought to be determined on the basis of quantification and tool-based mediation between the subject-as-researcher and the object of inquiry (Daston and Galison, 2007; Huniche and Sørensen, 2019), claims that also variously appeared in psychology (Danziger, 1990; Baker, 1992). By means of our objectifying tools, we have increasingly broken up the world into smaller and smaller parts (Gozli and Deng, 2018), a process that has been accompanied by an increased fragmentation of academic and intellectual disciplines (Cornejo, 2017). Huxley (1937, p.276) wrote that “intensive specialization tends to reduce each branch of science to a condition almost approaching meaninglessness” and that there “are many men of science who are actually proud of this state of things. Specialized meaninglessness has come to be regarded, in certain circles, as a kind of hall-mark of true science.” While the social sciences, including psychology, are often thought of as latecomers to this process, social scientists in fact played an important role in the transformation of scientific methodology into a scientific philosophy (i.e., scientism), as seen in the development of influential social theory (e.g., Auguste Comte) and even the development of formal methods of quantification and statistical testing (e.g., Francis Galton, Karl Pearson, and Charles Spearman; Bury, 1920; Sheen, 1934/2019; Scriven, 1966). Over time, science came to be increasingly thought of as fundamentally separate and independent from other epistemologies (Daston and Galison, 2007). Mainstream psychology came to primarily conduct *method-driven research* (Danziger, 1990; Huniche and Sørensen, 2019). Steinmetz (2005) has argued that not only does psychology have a close relationship with positivism but also some subfields such as social psychology are openly hostile to non-positivistic approaches. The degree to which positivism has been absorbed into psychology, he argues, makes it difficult to identify subfields that are truly free from it: “Even to locate a frontier between positivism and non-positivism in the fields concerned with the psychic, then, we are best advised to move beyond psychology into psychoanalysis” (p.12). Interestingly, this “frontier” implies that even within Freud’s psychoanalysis, there is a considerable degree of positivism (Elliot, 2005). What is more, most (but certainly not all) criticisms of psychoanalysis are based on various forms of positivism. For example, while Karl Popper may have been a vocal critic of *logical positivism*, his argument that psychoanalysis is essentially unfalsifiable is itself a positivistic position, although he did not himself like that term (Steinmetz, 2005). Popper largely rejected an inductionistic approach to science (1960); hence, his clash with the Vienna Circle and with what is often thought of as classical positivism. Within his hypothetico–deductive model of science, his deductivistic realism even makes room for imagination, fantasy, metaphysics, or guesswork as the starting point of scientific investigation. However, for Popper, these positions are only tenable to the extent that they be translated into empirically falsifiable scientific theories (Maxwell, 2017); hence, his line of thinking, as well as various other forms of hypothesis testing and probabilistic claims within psychology can be thought of as falling into the broad camp of positivism (and thus, he was attacked as a positivist by the Frankfurter School). Similarly, Popper believed intertheoretical translations to be of importance for the development of science, as it was only in this way that a new theory could be shown to be more falsifiable, but less falsified, than its predecessors (Laudan, 1996). Popper’s positivism can be seen all the more when contrasted with the thinking of Kuhn, whose “relativism about standards is the exact counterpoint of Popper’s methodological conventionalism” (Laudan, 1996, p.16). Nevertheless, although we are making use of a broad, inclusive, and rather simplistic understanding of positivism in this short piece, it needs to be clearly acknowledged that, like postmodernism, positivism is not a singular, homogenous philosophy, but rather speaks to a very diverse set of positions and practices (Kołakowski, 1968; Feichtigner et al., 2018). Mill’s (1865/2005, p.1) 19th century observation on the use of this label is further illustrative of this point: “more than one thinker who never called himself or his opinions by those appellations, and carefully guarded himself against being confounded with those who did, finds himself, sometimes to his displeasure, though generally by a tolerably correct instinct, classed with Positivists, and assailed as a Positivist.”^1^

While a simplified version of positivistic science has come to be thought of as a philosophy within our everyday understanding of knowledge, most scientists acknowledge the metaphysical nature of such a position. Thus, most scientists have come, at least implicitly, to agree with Huxley’s assertion, despite the claims of early positivists like Auguste Comte, that it “is impossible to live without metaphysic” (1937, p.252). To say that we should “let the data speak for themselves” contains the belief that *these* data are important, that they have something to say, and that it is worth hearing them out. However, most people’s understanding of science and matters of methods or metaphysics tend to be on precisely such a “bird’s eye” level, and the view of knowledge as being based on value-free, “objective” science is widespread (Aeschliman, 1983; Porter, 1996). “Decide on the basis of facts alone” became a mantra in modern times, and it remains so for many today, even among research psychologists. The continued dominance of positivism within psychology (Steinmetz, 2005), both as method and philosophy, shows that such thinking is widespread among social scientists who are aware of postmodern objections to positivistic epistemology. While various fields have gone through a wave (or waves) of intense empirical materialism, followed by periods of distancing from it, much of mainstream psychology appears to remain largely trapped in what Max Weber called the “iron cage of rationality,” whereby not only is validity determined on the basis of quantification and empirical measurement, manipulation, and control, but also all other forms of knowledge are deemed secondary, if not irrelevant (Danziger, 1990; Valsiner, 2012). Put even more plainly and at the risk of oversimplification—we remain impressed by numbers. The bittersweet humor of the aphorism that “90% of statistics are made up on the spot” speaks not to the problem of statistics or quantification *per se*, but to the widespread, exaggerated, and even problematic nature of their persuasive power (Porter, 1996).

## Mad as a Hatter: The “Madness” of Positivism and of Postmodern Responses

A flippant person has asked why we say, “As mad as a hatter.” A more flippant person might answer that a hatter is mad because he has to measure the human head. (Chesterton, 1908/2015, p.7)

Extreme positivism has indeed led to various forms of “madness,” but so too has the postmodern response to it. This is what led Laudan to call postmodern thinkers “the *new* crazies” (1996, p.3, italics added), a phrase which suggests the madness of both groups. As the general confusion sewn by postmodernism is well-known and widely discussed (e.g., Sokol and Bricmont, 1998; Valsiner, 2009; Hicks, 2011; Scruton, 2019), we will devote more time here to the madness of positivism. We will now briefly examine an illustrative example of the madness that can arise from the positivistic project.

Singer (2013), a moral philosopher and animal rights activist, made a telling utilitarian argument on the basis of positivistic *methods* turned positivistic *philosophy* for “effective altruism” (elsewhere called “outcome-based funding”). Effective altruism asserts that the quality of a charity can be determined on the basis of the number of quantifiable outcome units that result from each monetary unit invested in the given charity. Singer suggests that since it costs on average 40,000 United States dollars to raise and train a seeing-eye dog for a single blind American, but that the same amount of money could pay for operations that would allow between 400 and 2,000 people to regain their eyesight in the developing world (e.g., to treat glaucoma or cataracts), we should all redirect our donations from the first to the second kind of charity. Once the utilitarian ground has been laid, Singer points to the suffering, not of 2,000 sentient beings, but of literally billions of animals on the planet due to meat production, deforestation, and other forms of mistreatment. If we are counting, that number is certainly impressive and indeed heartbreaking, so perhaps that cause is an even better, more deserving recipient of our charitable donations. Singer does not stop there. Just as one blind person can be trumped by 2,000 blind people, and 2,000 blind people can be trumped by billions of suffering animals, so too can billions of suffering animals be trumped by the potential destruction of the entire planet along with all the creatures, big and small, living thereon. Thus, suggests Singer, perhaps we should be giving our charitable donations to the development of technologies that would shoot potentially Earth-ending asteroids out of the sky (or rather, shoot them to smithereens before they get that close). Rather than donating to afterschool programs for at-risk youth, suicide prevention hotlines, or cultural centers for the elderly, the quantification inherent in “effective altruism” would have us send checks to the space program. Here, decisions are made on the basis of mathematical calculation; responsibility lies in the tools, rather than in our own hands.

Postmodernism is currently one of the main voices in the social sciences that stands actively against the overly restrictive *Menschenbild* of positivism (Hicks, 2011). Much like the proliferation of new Protestant denominations after Catholic authority had been challenged, the notion of postmodernism can be thought of as an umbrella term that includes a large, and seemingly ever larger, number of different schools of thought (Steinmetz, 2005). Collectively, postmodernism is a set of inherently “critical, strategic and rhetorical practices […used…] to destabilize other concepts such as presence, identity, historical progress, epistemic certainty, and the univocity of meaning” (Aylesworth, 2015). An attempt to define postmodernism, or even to explore it to any satisfactory degree, is well beyond the scope of the current piece. After all, “[t]hat postmodernism is indefinable is a truism” (Aylesworth, 2015). Postmodern thinkers have done much to shake the overly restrictive foundations of positivism, often by means of the intuitively unsatisfying nature of positivism, and they certainly have captured the imagination of many a social scientist and “layperson” alike (Sokol and Bricmont, 1998; Hicks, 2011; Scruton, 2019). Postmodernism has time and again illustrated how our lived experience in effect simply slips through the bars of positivism’s methodological and epistemological iron cage. It has defended a wider scope of epistemological validity than that offered by positivistic reductionism alone (Steinmetz, 2005). However, postmodernism’s primarily deconstructive nature remains inherently wedded to positivism, as its negation. Thus, while postmodernism rightly challenges positivism’s expansion of usefully reductionistic method into overly restrictive philosophy, it has been unable to offer satisfying methodological or philosophical alternatives (Sokol and Bricmont, 1998; Hicks, 2011; Scruton, 2019). “[P]ostpositivism is an intellectual failure. The arguments on its behalf are dubious and question-begging. Still worse, it has sustained virtually no positive program of research” (Laudan, 1996 p.5). Within psychology, “the post-modernism avalanche has been the replacement of inquiry by an epistemological battlefield” (Valsiner, 2009, p.2). However, the failure of postmodernism is not necessarily *sui generis*:

what proved to be the undoing of postpositivism was not its departures from the positivist orthodoxy that preceded it. Rather, what has doomed postpositivism to amount to little more than a hiccup in the history of epistemology is the fact that it has carried to their natural conclusion several tendencies indigenous to positivism itself—tendencies that, once one sees their full spelling out, turn out to be wholly self-defeating. (Laudan, 1996, p.6)

Thus, postmodernism’s “barbaric vagueness” can itself be understood as arising in large part from earlier attempts to “measure the head” (Tolman, 1992). It is therefore to early criticisms of positivism that we now turn to.

## *Sapientia* as an Early Objection to Positivism

Challenges to the kinds of materialistic reductionism and quantification seen above are nothing new. For example, in contrast to the rationality of the Enlightenment, the likes of Vico and Herder asserted the fundamental importance of what Theodor Lipps later called empathetic *Einfühlung*, whereby we “feel into” the lives of qualitatively different others (Berlin, 1976). Similarly, as soon as psychology and sociology appeared at the university as independent disciplines at the turn of the 20th century, social scientists struggled to assert the fledgling fields’ independence of philosophy on the basis of empirical research, while being simultaneously aware of the very serious limitations of reductionism (usually quantification and control), causal explanation, and prediction (Bruner, 1990). Even the founder of the first psychology laboratory in 1879, Wilhelm Wundt, became concerned with the overly positivistic turn the field was taking and thus attempted to promote a more historically oriented, interpretive cultural psychology (*Völkerpsychologie*) as a form of counterbalance (Bruner, 1990; Valsiner, 2012). Similar to Wundt, Max Weber thought that our ability to predict human behavior was improved by the social sciences, but that it was also limited. He believed the best we could achieve in combining scientific methods and human variability was “adequate causality,” an approach that still very much describes the actual practices of statistical analysis in the social sciences today, even if hidden behind more strongly positivistic language (Tolman, 1992; Ringer, 2004). While he believed in the value of what he called the “ethic of responsibility” (*Verantwortungsethik*), whereby meaning is determined on the basis of causal relations in the empirical world, he was equally convinced of the need for the “ethic of conviction” (*Gesinnungsethik*) whereby the ends of human action, in the form of non-quantifiable values and meanings, must necessarily guide human life (Weber 1903–06/1975, p.192). Thus, according to Weber, the “social sciences, which are strictly empirical sciences, are the least fitted to presume to save the individual the difficulty of making a choice” (1949, p.19). Even the practicing scientist cannot escape living in a world that extends beyond the reductionistic horizon of positivism; “It can never be the task of an empirical science to provide binding norms and ideals from which directions for immediate practical activity can be derived” (Weber, 1949, p.52).

Perhaps the nearest we can get to expressing it is to say this: that his mind [the follower of scientism] moves in a perfect but narrow circle. A small circle is quite as infinite as a large circle; but, though it is quite as infinite, it is not so large. (Chesterton, 1908/2015, p.8)

This short quotation wonderfully captures both the tremendously broad, and simultaneously limited, scope of positivism. The power of the scientific method for better understanding and manipulating the material world is undeniable. At the same time, in describing positivistic science as infinite but nevertheless “not so large” as *sapientia*, Chesterton uses the language of quantification to poetically evoke that which cannot be quantified, that which lies beyond even the infinite reach of science.

In the modern world, it has become increasingly difficult to speak about wisdom because we have indeed become trapped in what Max Weber called the “iron cage” of rationality. We have come to expect small, bit-sized slices of information to satisfy our search for knowledge, and to save us the difficulty of making discriminating judgments (Scruton, 2007). This can be clearly seen in the example of Singer’s utilitarian assessment of charitable donations. Broadly speaking, this is a truly daunting problem. How can we consciously and conscientiously reflect on the strengths and weaknesses of positivistic epistemology when that has become the dominant epistemological language we have come to speak? Wittgenstein (1953) expressed something similar when he wrote: “The existence of the experimental method makes us think we have the means of solving the problems which trouble us; though problem and method pass one another by” (p.232). So as to break free of such positivistic language, advocates of *sapientia* have generally spoken in images or pictures rather than facts or data, in terms of qualities rather than quantities, in the language of poetry rather than prose, with the aid of judgment-provoking questions rather than unequivocal answers. In opposing the rigidity of positivism, thinkers such as G. K. Chesterton, C. S. Lewis, and L. Wittgenstein, generally wrote in a fantastical, poetic style, and often even in the language of fantasy or poetry themselves. To be clear, these thinkers were not against science, in the form of positivism-as-method. In fact, quite the opposite was generally the case. However, they were against *scientism*; the worship of a powerful method that, by default and design, constructively impoverishes our view of life and our *Menschenbild*.

This is the paradox of imagination in science, that it has for its aim the impoverishment of imagination. By that outrageous phrase, I mean that the highest flight of scientific imagination is to weed out the proliferation of new ideas. In science, the grand view is a miserly view, and a rich model of the universe is one which is as poor as possible in hypotheses. (Bronowski, 1964, p.46)

The power of this impoverishment has been historically connected to Hume’s separation of “is” from “ought,” that is, the separation of inductive observations from normative positions (Brinkman, 2019). This is precisely the split that Weber and others (e.g., Popper) both acknowledged and found problematic. While some have argued the division between “matters of fact” and “matters of concern” to be largely artificial (Knorr-Cetina, 1991; Latour, 2004), both the power and pull of the scientific method is all but undeniable, and it echoes even in postmodernism (i.e., as its rebuttal). From the point of view of *sapientia*, it is not that scientific reductionism, causal explanations, or attempts at prediction are problematic in themselves. In fact, they are powerful and incredibly useful tools. Rather, the problem is that they have come to constrict our field of vision, leaving us with a myopic focus on the tools before us rather than a broader view of the wider world. Just as one should not forget the house for the hammer and the nails, one should not confuse the house with a home. In the words of C. K. Lewis, “second things suffer when put first” (in Aeschliman, 1983, p.33). This is similar to what Ortega y Gasset, 1948/1968, p.19) called the dehumanizing effect of “inhuman inversion” and what Polanyi (1958) called “moral inversion.” If we understand science as a tool—not the only tool at our disposal, although a very important one—for the study of our lives and our world, we will be able to once again (re)focus on the important questions, rather than the questionnaires, that are driving our investigations.

Given our strong attachment to positivism in psychology, we may find ourselves still wondering at this point what exactly *sapientia* is, which is to say that we want a clear, positivistic definition. While we can confidently assert that the search for wisdom is roughly as old as humankind and that this search is found across cultures (Speer, 2005; Staudinger and Glück, 2011), singular definitions are inherently unsatisfying. Attempts to unilaterally define the notion belies the pull of positivism and the processes of increasing rationalization (Weber, 1949; Harrison, 2015). Some have even argued that “wisdom is the prototype of the class of psychological phenomena that by definition are unapproachable and unexplainable through scientific analysis” and that “to make wisdom transparent and to transform it into a subject matter of public knowledge and scientific debate is bound to change its basic foundation” (Baltes and Smith, 1990, p.89). This challenge aside, there have been considerable efforts by psychologists to conceptualize and operationalize wisdom so as to turn it into a measurable variable for empirical study (for overviews see Baltes and Smith, 2008; Staudinger and Glück, 2011; Ferrari and Weststrate, 2012; Bangen et al., 2013). These efforts are certainly laudable and have yielded valuable insights into the nature of our psychological lives. Nevertheless, to assume that this line of empirical research can capture the richness of wisdom is to misunderstand the issue at hand (Midgley, 1989; Maxwell, 2007). This is an example of Bronowski’s “impoverishment of imagination” whereby we meaningfully and usefully make sense out of an otherwise essentially overwhelming phenomenon, forgetting in the process how much is necessarily lost in translation. While this approach can be valuable, it is also precisely what allows postmodernism to meaningfully object; in this case arguing that wisdom has been too narrowly and rigidly defined. However, in moving away from the ostensible clarity of empirically grounded conceptualizations and operationalizations, postmodernism turns quickly into a form of “anything goes” that renders the concept subjective to the point of meaninglessness. While positivism pushes figures and facts to the point of fault, postmodernism does so with the world of subjective feelings. By contrast, and at the risk of oversimplification, the approach of *sapientia* stresses neither figures nor feelings, but the elevating aspect of fantasy. It does not attempt to definitively solve the challenges before us (as do the positivists), nor does it attempt in essence to deny the existence of those challenges (as do the postmodernists). Rather, it recognizes the perennial relevance of the questions. Within clinical psychology, for example, while positivistic approaches would attempt to identify answers to questions of mental health, and postmodern approaches would illustrate the relative and subjective nature of both the questions and the answers, *sapientia* would remind us of the importance of reflecting again and again on the questions, e.g., what is mental health?

Thus, *sapientia* escapes clear, fixed definition. Like the haiku, it breaks free of representation, be it in numbers, words, linearity, circularity, and sequentiality, etc., while simultaneously avoiding the “anything goes” aspect of postmodernism. This understanding of *sapientia* is metaphorically explained by G. K. Chesterton (1908/2015 p.14) as follows: “The one created thing which we cannot look at is the one thing in the light of which we look at everything. Like the sun at noonday, mysticism explains everything else by the blaze of its own victorious invisibility.” […] “But the circle of the moon is as clear and unmistakable, as recurrent and inevitable, as the circle of Euclid on a blackboard. For the moon is utterly reasonable; and the moon is the mother of lunatics and has given to them all her name.” Below we briefly review a few examples of how *sapientia* can reassert itself when we draw our attention back from a scientistic attachment to method, or what Gordon Allport called “methodolatry” (cited in Bruner, 1990, p.xi).

### The Reinstatement of the Individual

The more science-as-method became science-as-philosophy, the less relevant became the individual to our overall intellectual pursuit in psychology. The reductionistic materialism of positivism has tended to reduce the attention paid to the individual by placing an increased focus on the aggregate. Danziger (1990) referred to this as the “triumph of the aggregate.” Measures of central tendency are highlighted and outliers ignored or removed. This can allow us to see general principles beyond individual data points, and it is from this that science generally gains its power. While ideographic research has had a long and ongoing influence on the development of the sciences, including psychology, it is generally only considered to constitute scientific knowledge once it has been extended beyond the individual (Salvatore and Valsiner, 2010). Numerous breakthroughs in psychology occurred in precisely this manner (e.g., memory research with patient “H.M.”), as has been the case in other fields as well (e.g., studying individual planets or other cosmic bodies that are not easily replicable).

The strength of this epistemology-of-the-aggregate is also reflected in postmodernism in at least two interesting ways. On the one hand, as a reaction to positivism, postmodernism explicitly attempts to negate the epistemological certainty it affords. Thus, aggregate-level data are largely rejected, and the lion’s share of attention within postmodern psychology is received by the individual case, especially the explicitly idiosyncratic case that cannot be readily linked into a larger collective or calculated into averages (such cases can, of course, also be on the group level, for example, by focusing on a particular collective). However, lest postmodernism fall into epistemic certainty on the individual level, following the exploration of a particular case, one often sees assertions of wider polyvalence, whereby the singular voice in question is but one among many such voices. In the words of Ernest E. Boesch, “a broom is a broom is a broom,” by which he meant there are many different ways of seeing even a single, simple object (cited in Straub and Weidemann, 2007). On the other hand, despite such attempts to reject the modern epistemology-of-the-aggregate, postmodernism often sneaks such positivistic thinking in by the back door. For example, even when acknowledging the researcher’s necessarily subjective position, postmodern psychology attempts to retain some sense of (“scientific”) objectivity. This often takes the form of tool- or method-based distancing from the object of study, as such distance is understood—in line with the positivistic notion of objectivity (Porter, 1996; Daston and Galison, 2007)—to assure the validity of their truth claims about those same objects. For example, such notions as reaching the saturation point across interviews or setting a concrete number of interview subjects “required” to reach validity now seen in many journals, are in fact doing epistemology-of-the-aggregate without numbers (Holzkamp, 2013; Sousa, 2014; Huniche and Sørensen, 2019). Such methods are in effect different versions of letting the “data speaking for themselves”; the conclusions are being drawn from the objects of study via presumably “neutral” tools, and they are assumed to not be coming directly from the researcher, while they remain in many cases deeply method drive (Huniche and Sørensen, 2019). Thus, the ostensible openness and judgment-free position of the postmodern researcher, similar to the tool-based or method-based mediation of the natural sciences and positivistic psychology, distance the researcher from their research object, suggest the data to speak for themselves, and allow for claims of objectivity. This is psychology’s version of what Whitehead (1920) called the bifurcation of nature, whereby the observer’s tools allow them to objectively observe the observed. Thus, postmodernism tends to involve the explicit denial of the epistemology-of-the-aggregate, while simultaneously implicitly retaining it, although in a more subtle form.

Advocates of *sapientia* are not necessarily concerned with aggregate data, but by what Passmore (1978) called the “de-anthropomorphization” of human beings that can arise when the aggregate is valued above the individual. Historian Arnold Toynbee (1972/2015) was concerned by the “fanatical worship of collective human power,” something that C. K. Lewis referred to as “that hideous strength” (Aeschliman, 1983, p.27). Incidentally, it is not surprising that psychology’s maturity as a scientific and academic discipline at the start of the 20th century was marked by its close association with eugenics, perhaps the paradigmatic example of valuing the collective over the individual (Yakushko, 2019). A foreshadowing of this can be heard a century earlier in Comte’s lament regarding “the perennial Western malady, the revolt of the individual against the species” (cited in Hayek, 1944/2007, p.70). Advocates of *sapientia* thus share postmodernism’s assertion of the individual in abstraction from the collective, the promotion of the individual data point apart from the aggregate, in advocating for the outlier rather than the mean. However, *sapientia* differs from postmodernism by virtue of its simultaneous embrace of science *as method*, including the aggregate-level insights that come with it. What is more, *sapientia* asserts the value of the individual and the subjective as metaphysical values, a claim that postmodernism, in its general eschewal of epistemological certainty, would tend to avoid.

### The Notion of Progress

Positivism is not only committed to the “infinite but narrow” circle of various forms of reductionism, but it is also deeply wedded to the notion of linear, unidirectional progress (Bury, 1920; Löwith, 1949). Positivists, including many psychologists today, believe (either explicitly or implicitly) that science marches progressively forward, and that with each new step we are that much closer to building a better, and perhaps even more perfect, world. The evolution of this particular understanding of linear, material progress arose over millennia, from the cyclical understanding of history of the Greeks and Romans to the Christian understanding of history that grafted otherworldliness (i.e., of salvation in the afterlife) onto Judaic historical linearity (Löwith, 1949). Positivism arose from this linear understanding of history, but rejects the notion of otherworldly hope, placing its telos in the material world. The hope of positivism lies in material progress and the (often implicit) belief in the perfectibility of the world. Within modern positivistic thinking, this understanding of progress has been further supported by numerous processes of conceptual reframing, such as the tendency to retrospectively (re)define “successes” (i.e., past positions that are supported today) as part of science, and “failures” (i.e., previously held positions that are rejected today) as unscientific (Sismondo, 1996). In this way, science appears to be always moving forward and on the right path.

While art history is certainly a serious academic discipline, few historians of art would argue that art has advanced in a linear manner over the centuries, and even fewer would profess a faith in the eternal, progressive march forward of the arts. Beauty has not gotten more beautiful, nor has our understanding of beauty been progressively improving. However, many scholars working in the natural sciences, such as chemistry or physics, would make what are at heart largely positivistic arguments regarding the evolution of their fields (even though these arguments would be more nuanced today than pure, classic positivism). The scientism and methodolatry of psychology encourage a similar understanding of the field as progressing in a linear fashion. Empirical research in psychology, in the form of “neat little studies” (Bruner, 1990, p.xi) is expected to explicitly build, step-by-step, toward an ever-better understanding of human psychological processes. By default, and all things being equal, that which we know today is thought to be better than that which we knew yesterday, but inherently, an impoverishment of what we will know tomorrow.

With its focus on empirical research within “neat little studies” and on the “publish-or-parish” model of career development (and survival), contemporary psychology has witnessed an exponential growth of data collection. Within psychology, the rate of data collection has far outpaced the development of theory (Valsiner, 2014), reminding us of Percy Williams Bridgman’s statement regarding mathematics: “As at present constructed, mathematics reminds one of the loquacious and not always coherent orator, who was said to be able to set his mouth going and go off and leave it” (cited in Sheen, 1934/2019, p.69). In addition to the numerous practical reasons for this development, one of the additional factors implicitly underlying it and implicitly justifying it is the positivistic belief in the forward march of progress on the basis of empirical data. The more data, the better. We have come to assume that the accumulation of facts equals the accumulation of knowledge. However, “Facts as facts do not make scientific knowledge” – “Experiments may abound, but there is no necessary increase in knowledge” (Sheen, 1934/2019, pp.60–61).

To the extent that postmodernism undercuts epistemic certainty, the notion of unilinear progress is an impossibility. In the face of multiple truths, not only is forward motion impossible to identify, but so too is a singular, “correct” path along which we may travel. Postmodernism therefore rejects the utopianism of positivism, and its understanding of polysemy often even challenges singular claims to progress. Like postmodernism, *sapientia* is not wedded to such a unidirectional notion of progress as is positivism. Within *sapientia*, the choices of today are not understood to be necessarily better than the ones of yesterday, and there is no necessary link between wisdom gained and the development of a better world. By seeing method as method, and not philosophy, *sapientia* promotes reflection on questions that are long-lasting if not eternal, even as particular puzzles about the material world might be solved. For example, even as we unravel many a mystery regarding particular issues of mental health (e.g., treating syphilitic dementia with penicillin), the question of what mental health is remains. Science is uniquely powerful for solving particular puzzles related to the material world, but it does not answer the fundamental questions of life. By shifting the telos back from the material world to broader metaphysical questions, claims of unilinear progress and utopian visions of tomorrow become more difficult to sustain. By the same logic, *sapientia* would not automatically subscribe to the assumed value of increasing empirical data collection within more and more “neat little studies.” However, unlike postmodernism, *sapientia* asserts the value of the larger questions, not because they can be unilaterally answered as asserted by the positivist, but because they give life a simultaneously perennial and variable directionality that is generally undercut in postmodernism. This point will become clearer when we examine contradictions below.

### Contradictions

The positivistic understanding of progress generally implies a rejection of contradictions. If two scientific claims contradict each other, something is amiss. Of course, in practice, scientists can come to contradictory conclusions for any number of reasons, and in fact, such tension lies at the heart of the scientific enterprise itself. Nevertheless, the power of science comes from its ability to make distinctions between competing theories and to explain or otherwise reconcile differences in the data. This is arguably broadly similar to the galvanizing and creative role of conflict seen in more specific schools of thought, such as conflict theory or Hegelian dualism, whereby social progress is made not on the basis of contradictions or conflicts themselves, by in the form of some sort of resolution (which is incidentally a nice example of how the postmodern search for conflict is deeply rooted to the positivistic search for resolution, Laudan, 1996). Within clinical and developmental psychology, this often appears in the form of various “crises,” which lead to psychological growth (for an exploration of the “either-or” choice between objectivism and subjectivism in psychology, see Mos and Boodt, 1992).

That the need to resolve contradictions lies at the heart of the positivistic enterprise can be clearly seen in mainstream, quantitative psychology when experiments produce inconsistent or even contradictory findings. Researchers often work very hard, if not to definitively resolve them, then to explain them away by the addition of yet more variables; thereby kicking the proverbial can further down the road. An example of this can be seen in mainstream psychological research on power. Embedded within a larger positivistic research paradigm (including reductionistic conceptualizations and operationalizations, assumptions of variable control, the separation of variables, claims of causality, and replicability, etc.), high levels of power have been reported to make people lazy information processors, relying on previously held heuristics, and less flexible in the face of new information; however, other studies have found high levels of power to make people efficient information processors, relying less on previously held heuristic rules, and being more flexible in the face of new information (Guinote, 2015; Mazur, 2015). Such contradictory findings led to the formulation of the *situated theory of power* (Guinote, 2010), whereby variable, and even contradictory, information processing strategies are possible at high levels of power… depending on other variables (e.g., differences in motivation, goals, and situational factors). In light of such “variable buttressing,” whereby new independent variables are added to hold up the presumed causal effects of other variables, it is not surprising that the main concept of interest itself slips further and further away. From variable buttressing emerge such claims as the following: “It may be less useful to seek a unified definition of power than to focus on systematic mapping of how the effects of power covary with the kind of power studied; that is, perhaps we are always consigned to study just one limited aspect of power at a time, but we can do so deliberately and explicitly, using multiple perspectives and approaches in programmatic research” (Overbeck, 2010, p.32). Here we see a “three cup trick” at work, whereby the addition of yet more variables distracts us from the main point of interest; the definition of the construct (here “power”) vanishes, while these definitional problems somehow do not exist for subcategories of the construct (here “various kinds of power”) precisely because they lead to measurably different outcomes. Thus, within such positivistic research programs, the *definitional nature* of key concepts becomes their *functions* within larger causal chains (Mazur, 2015). This belief in the progressive value of adding rules to further explain previous rules, which were themselves expansions of previous rules, has been called the “additive fallacy” (Mazur, 2015). Interestingly, this process of smoothing out contradictions can be linked back to the drive for, and assumed value of, increased data collection and the linear progression of knowledge discussed earlier.

Postmodernism rejects this additive approach to knowledge and objects to this smoothing out of the rough edges of our lives in the name of singular explanations. Being reactionary in nature (i.e., “against” positivism), postmodernism actually highlights and promotes precisely such rough edges. In other words, postmodern thought is not so much an assertion of particular contradictions, but of contradiction itself. Within postmodernism, the contradiction is understood to be a tool to destabilize essentialized identities, the notion of historical progress, epistemic certainty, and the univocity of meaning. The postmodern voice is an “oppositional voice, a cry against the actual on behalf of the unknowable” (Scruton, 2019, p.15). Phenomenon and method are merged, and truth becomes indistinguishable from discourse. Sticking with the example of power, being “coextensive with the social body” (Foucault, 1980, p.142), power is understood in postmodernism as fluid and undefinable, but also ubiquitous. Opposition to power is inherently opposition to the social, particularly social stability, whatever that might mean in the given time and place.

Positivism is an attempt to remove contradictions, and postmodernism works to counteract those efforts. *Sapientia*, on the other hand, recognizes the insights gained by scientific discrimination (i.e., judging competing theories on the basis of data), while it also celebrates the contradictions of life—and it does so for their constructive, rather than deconstructive, value. In this tradition, contradictions, much like scientific tools, can be of epistemic value. Stated in the reverse, advocates of *sapientia* object to the reconciliation or resolution of contradictions seen in positivism (an objection shared with postmodernists), but they also object to the “contradiction-as-epistemic-uncertainty” found in postmodernism. *Sapientia* sees contradictions as a source of knowledge, as a way to break from the reductionistic rigidity of positivism. This particular celebration of contradictions has been called “bi-polar extremism” (Barron, 2004) within the Christian tradition, but it also finds expression in other religious, cultural, and historical contexts, such as the second century Mahayana Buddhist texts of Nāgārjuna (Garfield, 1995). The bipolar extremism of *sapientia* also finds expression in psychology, such as in Jungian archetypes (e.g., where each person contains light and shadow, male and female) or more recently in the notion of catalyzation within cultural psychology (Valsiner, 2014). Within *sapientia*, contradictions constitute paradoxes that inspire, not puzzles to be solved (as for the positivist) or perennial negations of truth at which we might throw up our hands in resignation or despair (as in postmodernism). The lover of *sapientia* “always cared more for truth than consistency. If he saw two truths that seemed to contradict each other, he would take the two truths and the contradiction along with them. His spiritual sight is stereoscopic, like his physical sight: he sees two different pictures at once and yet sees all the better for that” (Chesterton, 1908/2015, p.14). To stick with the metaphor of images, we can say that while positivism attempts to resolve the multistability of contradictions so that one truth wins out, and while postmodernism oscillates back and forth between the two *ad nauseam*, *sapientia* allows us to see both images at once (for a discussion of multistability see Mazur, 2019). With regard to power, perennial reflections on the nature of power lie at the heart of *sapientia*, and the questions that emerge are neither answered definitively away (as in positivism) nor deemed essentially subjective and unanswerable (as in postmodernism).

### Wonder at the Ordinary

Our tendency to be enamored by positivistic reductionism, including quantification, has in effect distracted our attention from the actual object(s) of interest. This point can be seen in the quantification of psychological phenomena. Gustaw Ichheiser expressed this general challenge of positivistic psychology as follows: “the higher the adequacy of a psychological description, the stronger, paradoxically, the inevitable impression that ‘nothing new’ was really presented” (1943, p.207). The overquantification of psychological phenomena seen in modern psychology can cause a form of amnesia whereby we forget what we were interested in the first place:

the psychologist may possess the knowledge about certain psychologically relevant facts as long as he is not acting in his capacity as a “psychologist.” But, paradoxically enough, he forgets, ignores, or neglects those facts as soon as he transforms himself into a psychological expert and in this role performs some scientific research. (Ichheiser, 1943, p.206)

To be clear, the point is not that quantification cannot bring with it important insights regarding the nature of our world and our lives, including psychological phenomena. In fact, *sapientia* would not necessarily oppose the use of any tools at our disposal to better understand our lives. What is more, science—even strongly positivistic aspects of science—can certainly inspire wonder. This wonder cannot only inspire scientists in their work, but also it can often lead them to understand science to be, in fact, the ultimate source of wonder. Writing in the areas of physiology and psychology, Emil (Harleß, 1851, pp.20–21) captures this sentiment beautifully when he argues how the wonder of the natural world can remain hidden from what he calls “the unarmed eye” (*das unbewaffnete Auge*; without scientific tools like the microscope): “We are amazed by the beauty and the regularity with which the delicate plant cells are aligned with each other—and we trample with indifference the leaves of grass that are built out of them.” However, the wonder such investigation can cause lies not in the numbers or the methods or even the objects of investigation themselves, but in us (Polanyi, 1958; Wagoner et al., 2017). An awareness of the humanity of wonder is reflected in the stereotyped presentations of strictly positivistic minds in popular culture, such as Sherlock Holmes or the characters Mr. Spock and Data from Star Trek, characters whose excessively analytical minds are marked by a reduction in human emotion, including the sense of wonder. By way of contrast, Holmes’ sidekick Dr. Watson is himself a scientist, but he is also deeply human, and he records the adventures of Sherlock Holmes precisely because of his sense of wonder at this uniquely overly analytical man. A similar contrast to the character of Spock is seen in the passionate character of Captain Kirk.

“And so we see that the poetry fades out of the problem and by the time the serious application of exact science begins we are left only with pointer readings” (Eddington, 1928/2012, p.252). It should not surprise us that the “pointer readings” of psychological research do not perfectly match the reality of our psychological lives. In fact, it should come as a great relief. As a response to positivism, *sapientia* not only redirects our attention to the ordinary, but reawakens our wonder at it. The ordinary not only constitutes the basic building blocks of our lives, but it is what makes our lives fantastic. As G. K. Chesterton put it, things that are common, like death or first love, are not necessarily commonplace; “Ordinary things are more valuable than extraordinary things; nay, they are more extraordinary” (1908/2015, p.26). Various schools of thought have emerged in psychology that constitute attempts to reinvigorate the study of those very topics that have been in decline during the ascent of positivistic thinking (i.e., various versions of “cultural psychology,” not to be confused with cross-cultural psychology; Bruner, 1990; Lonner and Hayes, 2007; Valsiner, 2014). While such schools of thought generally lie outside the mainstream of psychology, they address issues that lie at the heart of the field, such as the study of mind rather than just the brain, the study of creativity rather than just causality, and the study of historically and socially based processes rather than presumed universality. These smaller branches of psychology thus share this fundamental similarity with early objections to positivism on the basis of *sapientia*; by redirecting our attention away from positivistic methods, they attempt to reawaken our wonder at that which has come to be seen as “ordinary” or that is made “normal” based on statistical normality. Here again, we hear echoes of postmodernism’s objection to claims of normality (Scruton, 2019). However, unlike in postmodernism where the relativism of polyvalence curtails the depth and reach of wonder (as but one possible view), *sapientia* allows us to retain the object of wonder, precisely as an object of wonder.

## Discussion

Writing over a century ago, Georg Simmel argued that our increasing reliance on calculability and rationality (e.g., within the money-based economy) was having a significant impact on human psychology and social relations more broadly. This arose from:

the growing preponderance of the category of quantity over that of quality, or more precisely the tendency to dissolve quality into quantity, to remove the elements more and more from quality, to grant them only specific forms of motion and to interpret everything that is specifically, individually, and qualitatively determined as the more or less, the bigger or smaller, the wider or narrower, the more or less frequent of those colorless elements and awarenesses that are only accessible to numerical determination. (Simmel, 1978/2004, p.278)

For the likes of Simmel and Weber, the problem was not so much with quantification itself, but with its imperial tendencies. Our reliance on calculability and rationality was changing the way we think and act. Something similar has happened in the field of psychology. Quantification has not only come to dominate as a methodological tool, but it has come to color our thinking about psychological phenomena more broadly. Despite the best efforts of postmodern thinkers, mainstream psychology retains a largely modern, positivistic epistemology.

Rather than rejecting the tools afforded us by positivistic thought, *sapientia* challenges us to recognize them as just that, tools. They are limited and necessarily unable to grasp the entirety of our psychological lives. Positivism is powerful as method; however, it is problematic as philosophy. To return to a metaphor used earlier, positivistic epistemology can help us identify the tools and the methods of their use by which we may build a more solid house, but it cannot make it a home nor even help us to better understand what that might require. In other words, positivism cannot fully capture the psychosocial meaning-making processes that should constitute the core focus of psychology (Bruner, 1990). In the words of Gustaw Ichheiser:

We should not expect and demand that everything should be “proved.” To say it once more, social scientists should, in my opinion, not aspire to be as “scientific” and “exact” as physicists or mathematicians, but should *cheerfully* accept the fact that what they are doing belongs to the twilight zone between science and literature. (cited in Rudmin et al., 1987, p.171, italics added)

It has been argued that we can indeed *cheerfully* accept this state of affairs, and that in doing so, we will be able to once again see aspects of our psychosocial lives that have become obscured by the dominant positivistic epistemology, such as the importance of the individual and of subjective experience, the notion of *personal* progress in a complex world, the epistemic power of contradictions, and a childlike wonder at our world. Positivistic epistemology in psychology is certainly powerful, but it is also of limited use; however, the way to approach those shortcomings lies not in postmodernism. Rather, we should more fully recognize that as psychologists, we “are still drawing rich sustenance from our more distant, pre-positivist past” (Bruner, 1990, p.x).

## Author Contributions

The author confirms being the sole contributor of this work and has approved it for publication.

## Conflict of Interest

The author declares that the research was conducted in the absence of any commercial or financial relationships that could be construed as a potential conflict of interest.

## Footnotes

1 This was expressed more recently by Kołakowski (1968, p.1) thus: “Not all, however, who according to historians or critics profess the positivist doctrine, would agree to be classified under this heading.”
